# Metabolomic profiling identifies distinct phenotypes for ASS1 positive and negative GBM

**DOI:** 10.1186/s12885-018-4040-3

**Published:** 2018-02-08

**Authors:** Lina Mörén, Richard Perryman, Tim Crook, Julia K. Langer, Kevin Oneill, Nelofer Syed, Henrik Antti

**Affiliations:** 1 Department of Chemistry, Umeå University, SE 901 87 Umeå, Sweden; 2 John Fulcher Neuro-Oncology Laboratory, Imperial College London, London, UK; 3 St Luke’s Cancer Centre, Royal Surrey County Hospital, Guildford, Surrey UK

**Keywords:** Glioblastoma, Epigenetics, ASS1, Arginine, ADI-PEG20, Metabolomics, Chemometrics

## Abstract

**Background:**

Tumour cells have a high demand for arginine. However, a subset of glioblastomas has a defect in the arginine biosynthetic pathway due to epigenetic silencing of the rate limiting enzyme argininosuccinate synthetase (ASS1). These tumours are auxotrophic for arginine and susceptible to the arginine degrading enzyme, pegylated arginine deiminase (ADI-PEG20). Moreover, ASS1 deficient GBM have a worse prognosis compared to ASS1 positive tumours. Since altered tumour metabolism is one of the hallmarks of cancer we were interested to determine if these two subtypes exhibited different metabolic profiles that could allow for their non-invasive detection as well as unveil additional novel therapeutic opportunities.

**Methods:**

We looked for basal metabolic differences using one and two-dimensional gas chromatography-time-of-flight mass spectrometry (1D/2D GC-TOFMS) followed by targeted analysis of 29 amino acids using liquid chromatography-time-of-flight mass spectrometry (LC-TOFMS). We also looked for differences upon arginine deprivation in a single ASS1 negative and positive cell line (SNB19 and U87 respectively). The acquired data was evaluated by chemometric based bioinformatic methods.

**Results:**

Orthogonal partial least squares-discriminant analysis (OPLS-DA) of both the 1D and 2D GC-TOFMS data revealed significant systematic difference in metabolites between the two subgroups with ASS1 positive cells generally exhibiting an overall elevation of identified metabolites, including those involved in the arginine biosynthetic pathway. Pathway and network analysis of the metabolite profile show that ASS1 negative cells have altered arginine and citrulline metabolism as well as altered amino acid metabolism. As expected, we observed significant metabolite perturbations in ASS negative cells in response to ADI-PEG20 treatment.

**Conclusions:**

This study has highlighted significant differences in the metabolome of ASS1 negative and positive GBM which warrants further study to determine their diagnostic and therapeutic potential for the treatment of this devastating disease.

## Background

Glioblastoma (GBM) is the most common and most lethal primary brain tumour affecting adults of all ages. Despite improvements in imaging, surgical techniques, radiotherapy and chemotherapy the prognosis remains poor with a median overall survival typically around 12 months in optimally treated patients. This poor survival is attributed to the highly invasive nature of GBM, making complete surgical resection almost impossible resulting in tumour recurrence in most cases. In addition, these tumours exhibit a high degree of radio and chemo resistance [1, 2].

Extensive profiling of GBM has led to a greater understanding of the underlying biology of this disease. For example, the majority of genomic lesions identified to date lie in three core signalling pathways (receptor tyrosine kinase/RAS/phosphatidylinosintol 3 kinase (RTK/RAS/PI3K), p53 and retinoblastoma (RB) [3]. Hence aberrant signalling through these pathways is likely to be essential for the development of GBM. Furthermore, these studies have identified four distinct molecular subclasses of GBM based on the enrichment of specific molecular alterations (proneural, classical, mesenchymal and neural). Interestingly, these subclasses were shown to have different responses to standard therapies [4].

This wealth of information has led to the development of several molecularly targeted therapies for GBM, some of which have shown promise in preclinical and clinical settings. However, most have failed to show promise in improving outcomes and hence the standard of care for GBM patients remains the same [5, 6].

Since cancer cells have a high reliance on glucose and amino acids to support their increased growth rate, one strategy to target them is the removal of an essential metabolic resource. This strategy has been successfully employed for the treatment of acute lymphoid leukaemia where asparaginase is the standard therapy in combination with chemotherapy for this cancer [7, 8].

From the initial observation that mycoplasma infection can kill cancer cells and spare normal cells [9] and the subsequent discovery that this was due an arginine degrading enzyme found in mycoplasma, arginine deiminase (ADI) [10, 11], there has been an explosion in the use of arginine deprivation as a therapeutic strategy for numerous cancers.

Arginine is a nonessential amino acid that fuels an array of metabolic reactions including nitric oxide synthesis, polyamines and amino acids such as glutamine and proline, all of which are important regulators of cell growth and survival [12]. Arginine is synthesized from aspartate and citrulline by two closely coupled enzymes of the urea cycle, argininosuccinate synthetase (ASS) and argininosuccinate lyase (ASL) with the former being the rate limiting step [13]. Healthy adults predominantly obtain arginine from dietary intake and from intracellular protein degradation but can also synthesize it when required and the level of synthesis is sufficient to meet their energy demands [14]. Tumour cells due to their rewired metabolism have a greater requirement for arginine and make use of the extracellular pools [15, 16]. Cancers that have reduced expression of ASS/ASL and unable to synthesise arginine become highly dependent on these pools and therefore susceptible to arginine deprivation therapy using arginine degrading enzymes [12, 17]. Although the mechanism of ASS/ASL downregulation is not completely understood, there is strong evidence for both promoter methylation and hypoxia-inducible factor-1α mediated transcriptional repression in some cancers [18, 19] and further mechanisms are likely to exist. Two enzymes that are continually being evaluated for their effectiveness in degrading arginine are: ADI-PEG20 (a pegylated form of ADI to reduce immunogenicity in humans and extend half-life) and recombinant human arginase 1 [20–23]. ADI-PEG20 degrades arginine to citrulline and ammonia and arginase 1 degrades it to ornithine and urea. Since the first reports of arginine deprivation in melanoma [24] and hepatocellular carcinoma (HCC) [25], the list of cancer types that are amenable to this therapeutic strategy is constantly growing and includes prostate, breast, ovarian, lung, sarcoma and malignant pleural mesothelioma to name but a few including our own study in GBM [26, 27]. Since most of these studies have used ADI-PEG20 to degrade arginine, this enzyme has been extensively evaluated [28]. Many of these studies revealed mechanistic insights into the molecular effects of arginine deprivation in ASS deficient tumours identifying additional vulnerabilities prompting the use of other agents in combination with this strategy to achieve more effective killing. For example the use of TRAIL in mesothelioma [29], cisplatin in multiple tumour types [28], chloroquine in sarcomas [30] and 5FU in HCC [31].

In contrast, given the diverse role of arginine in numerous metabolic pathways there are far fewer studies investigating the metabolic effects of arginine deprivation and to our knowledge no studies have been performed in GBM. One such study includes our own study in bladder cancer where we observed ASS negative cells had increased uptake of thymidine which becomes suppressed upon ADI-PEG20 treatment. Since thymidine can be imaged by positron emission tomography, its reduced uptake can serve as a biomarker of response to therapy [32]. Similarly, Locke et al. [33] through their metabolomic analysis identified that ASS1 negative mesothelioma have a dependency for polyamine metabolism and that ADI-PEG20 decreases polyamine metabolites. Thus, this finding provides a dual synthetic lethal strategy for ASS negative mesothelioma with ADI-PEG20 and inhibition of polyamine synthesis. Another study by Kremer et al. [34] identified a potential synthetic lethal interaction with ADI-PEG20 and glutamine inhibition in sarcoma, as a consequence of discovering up-regulation of glutamine anerplerosis and serine biosynthesis upon ADI-PEG20 therapy. To our knowledge, there are no studies specifically looking at basal metabolic differences between ASS negative and positive GBM tumours.

Metabolic reprogramming manifested as altered nutrient uptake and use has been proposed to be a hallmark of cancer. Since this reprogramming is thought to be essential for rapid cancer cell proliferation, a metabolomic analysis of cancer metabolism will paint a broad picture of the altered pathways and their interactions with each other. Metabolomics is the profiling of metabolites within a cell, the levels of which integrate the effects of gene regulation, post-transcriptional regulation, pathway interactions, and environmental perturbations [35, 36]. Thus, this downstream synthesis of diverse signals ultimately makes metabolites and patterns thereof direct molecular readouts of cell status that reflect a meaningful physiological phenotype.

Combined with bioinformatic approaches that consider the multivariate interaction between multiple variables (i.e. chemometric bioinformatics), metabolomics can aid to detect patterns of metabolites as biomarkers (latent biomarkers) to better map and predict complex metabolic events [37, 38].

The present study was carried out to investigate our hypothesis that metabolomic analysis, represented here by 1D and 2D GC-TOFMS combined with chemometric bioinformatics, can discriminate between ASS1 positive and ASS1 negative GBM cell lines and potentially identify metabolic biomarkers for the non-invasive detection of these subtypes and unveil additional novel targets for their treatment. Novel treatment strategies are desperately needed as currently there are no effective therapies for this devastating tumour.

## Methods

### Cell culture

All GBM cell lines used in this study are negative for the IDH1 mutation (previously sequenced in our lab) and were obtained from ATCC. ASS negative (LN229, SNB19, GAMG) and ASS positive (U118, T98G, U87) GBM cell lines were plated into 6 replicate wells of a 6 well plate at a density of 1.5 × 10^5^ cells/well in 3 ml of DMEM or MEM media (T98G) containing 10% fetal calf serum (FCS). Supernatants and cell pellets were collected 48 h after plating and stored at − 80 °C until required. Control wells containing media alone were included for normalization purposes.

### ADI-PEG20 treatment

SNB19 and U87 cells, cultured in DMEM + 10% FBS and normal human astrocytes, cultured in speciality media provided by lonza were seeded in replicates (*n* = 12) at 8 × 10^4^ cells per well in 6-well dishes (Corning, NY, USA). 24 h post seeding, cells were washed with phosphate buffered saline (PBS) and cultured in the presence or absence of ADI-PEG20 (1 μg/ml) in media containing, 1 mM citrulline and 10% fetal FBS. ADI-PEG20 was added at the start of the experiment and no fresh media was added to any of the experimental plates before harvesting. ADI-PEG20 treated and untreated media (*n* = 3) was included for normalization purposes. 48 h after ADI-PEG20 treatment replicate samples for each condition (*n* = 3) were harvested, collecting both spent media and cells for GC-TOFMS metabolomic analysis. Additional replicates (*n* = 3) of each condition were collected for total cell count determination.

### Analytical strategy

In an attempt to cover a large proportion of the metabolome and to detect and identify overlapping compounds, samples were screened for metabolites using both 1D and 2D GC-TOFMS. The results obtained from this initial screen were verified by a targeted amino acid analysis using LC-TOFMS.

### Sample preparation

Frozen samples (supernatants and cell pellets) were thawed at room temperature and 900 μl of extraction solution (90% methanol, 10% water, 7 internal standards (Salicylic acid, myristic acid, hexadecanoic acid, cholesterol, succinic acid, glutamic acid och sucrose; 7 ng/μl)) were added to 100 μl of supernatant and to the cell pellet. Two tungsten beads were also added to the pellets. Samples were extracted using a MM301 vibration Mill (Retsch GmbH & Co. KG, Haan, Germany) for 2 min at 30 Hz and placed on ice for 2 h and centrifuged for 15 min at 14,000 rpm at 4 °C. 200 μl of the supernatants were transferred to vials and evaporated to complete dryness before being stored at − 80 °C. Before 1D and 2D GC-TOFMS analysis the samples were methoxymated with 30 μl of methoxyamine solution in pyridine (15 μg/μl) first at 70 °C for 1 h and then at room temperature for 16 h. Thereafter, the samples were trimethylsilylated with 30 μl of MSTFA at room temperature for 1 h before the addition of 30 μl of heptane (containing 0.5 μg of methyl stearate). Prior to analysis, the samples were randomized and analysed together with a series of n-alkanes (C_12_-C_32_) to allow retention indexes to be calculated.

For the ADI-PEG20 experiment, samples were randomized, defrosted at room temperature and vortexed. For the cell media fraction, 200 μl were transferred to individual microcentrifuge tubes. A quality control sample was prepared from a pool of each sample, and 200 μl were transferred to individual microcentrifuge tubes. 1.5 ml of methanol was added to each sample, mixed for 5 min and spun at 10,000 g for 10 min at 4 °C. 1.3 ml of supernatant was transferred to individual silylated glass tubes and evaporated to dryness at 40 °C under nitrogen gas in a concentration evaporator (Turbovap LV, Biotage, Uppsala, Sweden).

For the cellular fraction, 200 μl cell lysate was transferred to individual microcentrifuge tubes and 1.3 ml extraction mixture (water:MeOH [2:10]) was added, giving a final methanol concentration of 80% together with the cell lysate. Samples were vortexed for 5 min for cellular disruption and extracted on ice for 20 min, following centrifugation at 4 °C at 17,500 g for 10 min. 1.4 ml supernatant was transferred to silylated glass tubes and dried at 40 °C under nitrogen gas with a concentration evaporator.

Both individual and pooled standards were prepared, making a stock solution in water (10 mg/ml). Individual samples were diluted in methanol (0.005 mg/ml), and 100 μl were transferred to silylated glass tubes. Pooled standards were diluted to 300 μl water containing metabolite standards and 200 μl methanol.

### GC-TOFMS

One μl sample was injected splitless by an Agilent 7683 Series autosampler (Agilent, Atlanta, GA) into an Agilent 6980 GC equipped with a 10 m × 0.18 mm i.d. fused-silica capillary column chemically bonded with 0.18 μm DB5-MS stationary phase (J&W Scientific, Folsom, CA). The injector temperature was 270 °C. Helium was used as carrier gas with a constant flow rate of 1 ml/min. The purge time was 60 s at a purge flow rate of 20 ml/min and an equilibration time of 1 min per analysis. Initially, the column temperature was kept at 70 °C for 2 min and then increased to 320 °C at 30 °C/min, where it was kept for 2 min. The column effluent was introduced into the ion source of a Pegasus III TOFMS (Leco Corp., St Joseph, MI). The transfer line temperature was 250 °C and the ion source temperature 200 °C. Ions were generated by a 70 eV electron beam at a current of 2.0 mA. Masses were acquired from m/z 50 to 800 at a rate of 30 spectra/s, and the acceleration voltage was turned on after a solvent delay of 165 s.

The acquired data was exported to MATLAB 7.11.0 (R2014b) (Mathworks, Natick, MA) as NetCDF files. An in-house script was used for alignment, peak detection, mass spectrum deconvolution, mass spectra library search for identification and calculation of peak height/area. To identify the detected compounds, the mass spectral profiles and retention indices were compared to spectra in an in-house spectra library established at Swedish Metabolomics Centre (SMC) (www.swedishmetabolomicscentre.se).

For the ADI-PEG20 treatment experiment, samples were subjected to GC-TOFMS on a Pegasus GC-TOFMS (Leco Corp., St Joseph, MI, USA), connected with an Agilent 7890 gas chromatograph, using a fused silica capillary column (Agilent J&W Scientific), with 0.25 μm thickness and open split interface. Under helium as carrier gas, samples were injected, with constant temperatures for injection (220 °C), transfer (280 °C), ion source (250 °C). Primary oven temperatures were programmed at 70 °C for 0.2 min, raised to 270 °C for 5 min and further increased to 310 °C for 11 min. 70 eV electron beams were used for the ionization and masses were recorded from 40 to 600 m/z at a rate of 20 spectra/s with the detector voltage set at 1650 V.

Gas chromatograms were baseline corrected, de-convoluted, noise-reduced, smoothed, library matched and areas were calculated with ChromaTOF software (LecoCorp. v 4.4). Signal-to-noise ratio above 150 were analysed. Putative analyte identities were found by comparing MS spectra with RI of the US National Institute of Science and Technology (NIST) library, as well as the Fiehn library, Golm Metabolome Database, Human Metabolome Database and in-house databases.

### 2D GC-TOFMS

The samples were analysed on a Pegasus 4D (Leco Corp., St Joseph, MI, USA) equipped with an Agilent 6890 gas chromatograph (Agilent Technologies, Palo Alto, GA, USA), a secondary gas chromatograph oven, a quad-jet thermal modulator, and a time-of-flight mass spectrometer. Leco’s ChromaTOF software was used for setup and data acquisition. The column set used for the GCxGC separation was a polar BPX-50 (30 m × 0.25 mm × 0.25 μm; SGE, Ringwood, Australia) as first-dimension column and a non-polar VF-1MS (1.5 m × 0.15 mm × 0.15 μm; J&W Scientific Inc., Folsom, CA, USA) for the second-dimension column. Splitless injection of 1 μl sample aliquots was performed with an Agilent 7683B auto sampler at an injection temperature of 270 °C (2 respectively 5 pre/post-wash cycles were used with hexane). The purge time was 60 s with a rate of 20 ml/min and helium was used as carrier gas with a flow rate of 1 ml/min. The temperature program for the primary oven started with an initial temperature of 60 °C for 2 min, followed by a temperature increase of 4 °C/min up to 300 °C and where the temperature was held for 2 min. The secondary oven maintained the same temperature program but with an offset of + 15 °C compared to the primary oven. The modulation time was 5 s with a hot pulse time of 0.8 s and a 1.7 s cooling time between the stages. The MS transfer line had a temperature of 300 °C and the ion source 250 °C. 70 eV electron beams were used for the ionization and masses were recorded from 50 to 550 m/z at a rate of 100 spectra/s with detector voltage set at 1780 V.

Baseline correction, peak detection, mass spectrum deconvolution, mass spectra library search for identification and calculation of peak height/area was done in Leco’s ChromaTOF software. For peak picking a signal-to-noise ratio of 10 was used. The library search was performed against publicly available mass spectral libraries from US National Institute of Science and Technology (NIST) and from the Max Planck Institute in Golm [39] together with in-house libraries established at SMC. csv-files (comma-separated values) containing peak information for each of the samples was exported. The csv-files were imported into the data processing software Guineu (1.0.3 VTT; Espoo, Finland) [40] for alignment and filtering.

### Amino acid analysis

Derivatization of amino acids was achieved using the AccQ-Tag kit obtained from Waters (Millford, MA) as specified in the manufacturer’s instructions. An amino acid standard mixture including 29 amino acids was prepared and diluted into eight concentrations ranging from 20 pmol to 0.1 pmol. An internal standard, norvaline [10 pmol/μl] was added to each sample and the amino-acid standards mixture. The samples were analysed on a Waters Acquity UPLC system coupled to a Micromass LCT Premier mass spectrometer (Waters, Millford, MA) operated in W-mode. The acquired data was exported as NetCDF files. MATLAB 7.11.0 (R2014b) (Mathworks, Natick, MA) was used with an in-house script for alignment and extraction of the integrated peak area for each amino acid.

### Pattern recognition and statistical analysis

The data from the cell pellet and the cell supernatant from the different analytical methods were analysed separately. Prior to any data analysis, data from the cell medium was subtracted from the cell supernatant data to minimize the influence of the cell medium in further investigations. Data from the amino acid analysis data was normalized to the internal standard norvaline.

A chemometric bioinformatics approach using pattern recognition based on multivariate projection methods was used to detect systematic patterns in the data associated with ASS1 status and the corresponding response to ADI-PEG20 treatment. In this way patterns of co-varying metabolites can be detected and evaluated both predictively as latent biomarkers and mechanistically by comparing the involved metabolites to known biochemical pathways.

To obtain an initial overview of the systematic variation in the data and detect deviating samples (outliers), the data was analysed using principal component analysis (PCA) [41], an unsupervised multivariate projection method focusing on the maximum variation in the data (not shown). Thereafter, to investigate potential differences between ASS positive and ASS negative cell lines as well as to evaluate the effect of arginine deprivation therapy by ADI-PEG20, OPLS-DA [42] was used. OPLS-DA is a supervised multivariate analysis method where the systematic pattern differences between pre-defined classes in the data are examined. Variable selection was used to extract metabolites responsible for the separation in the calculated OPLS-DA models. Model weight values (w*), i.e. variable contribution values for the pre-defined sample class separations, were extracted and metabolites with low w*- values (|w*| < 0.05), i.e. variables unrelated to the class separation were discarded [43]. All OPLS-DA models were validated using cross-validation and *p*-values for the cross-validated model were calculated using ANOVA [44]. A univariate *p*-value for each metabolite was calculated using the Mann-Whitney U-test.

### Pathway and network analysis

Identified metabolites were subjected to pathway analysis using the software Ingenuity Pathway Analysis (IPA). Accession numbers of detected metabolites (HMDB, PubChem, and KEGG Identifiers) were listed in MS Excel and imported into IPA to map the canonical pathways and generate networks of interacting biological entities. Data were submitted as fold change values (ratios) between ASS positive and ASS negative cells. Comprehensive pathway and network analyses were performed. Downstream biological processes were scored in accordance to the ontology support using Ingenuity Knowledge Base (www.ingenuity.com).

## Results

### GC-TOFMS analysis reveals metabolic differences between ASS negative and ASS positive GBM

We previously demonstrated that ASS negative GBM (deficient in the arginine biosynthetic pathway) can be targeted by arginine deprivation therapy using ADI-PEG20 whereas ASS positive GBM are unaffected due to their ability to endogenously synthesize arginine [27]. (It is important to note that these cells were negative for the IDH1 mutation (previously screened in our lab), a feature that can significantly impact on the metabolome. In an attempt to identify additional metabolic vulnerabilities between these two subgroups of GBM, we profiled the metabolome of a panel of ASS negative (LN229, SNB19 and GAMG) and ASS positive (U118 and T98G) GBM cell lines using 1D and 2D GC-TOFMS. GBM cells were cultured for 48 h as described in materials and methods and both the cell supernatants and cell pellets were harvested for analysis.

Using 1D GC-TOFMS, we identified 76 and 83 unique metabolites in the supernatant and cell pellet respectively by comparing mass spectra and retention indices to existing and available compound libraries. In contrast, using 2D GC-TOFMS a greater number of peaks were retained in both fractions (815 in supernatant and 317 in cell pellet) after filtering in Guineu (1.0.3 VTT; Espoo, Finland). Of these peaks we could adequately identify 89 unique metabolites in the supernatants and 83 in cell pellets.

**Fig. 1:**
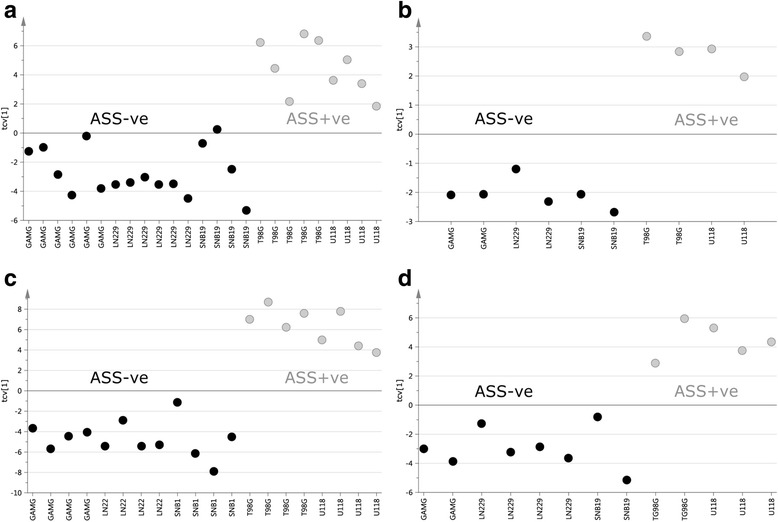
Cross-validated scores, first predictive score (tcv[1]), based on the final OPLS-DA models showing an almost complete separation of ASS+ve cell lines (T98G and U118; grey) and ASS-ve cell lines (GAMG, LN229 and SNB19; black) for (**a**) 1D GC-TOFMS data cell supernatant (*p* = 1.84*10^− 5^) and (**b**) 1D GC-TOFMS data cell pellet (*p* = 0.03) and (**c**) 2D GC-TOFMS data cell supernatant (*p* = 0.0007) and (**d**) 2D GC-TOFMS data cell pellet (*p* = 0.004)

The data generated using both 1D and 2D GC-TOFMS was subjected separately to multivariate analysis by means of OPLS-DA to model systematic differences in metabolite patterns between ASS positive and ASS negative cell lines. This supervised multivariate projection method allows for a division of the variation in the data into a predictive part which is related to a specified difference i.e. ASS1 status, and an orthogonal part which is unrelated to this difference. These variation sources can be overviewed and interpreted on the sample level in the OPLS-DA scores and on the variable level (contributing metabolite patterns) in the OPLS-DA loadings. This analysis revealed a clear systematic difference between ASS1 positive and ASS1 negative cell lines using both 1D and 2D GC-TOFMS data in both the cell pellet and supernatant (Fig. 1). Further analysis of the metabolites contributing to this difference revealed an elevation of those involved in the arginine biosynthetic pathway in ASS positive cells compared to ASS negative cells (Tables 1 and 2). Moreover, ANOVA of the cross validated model showed a statistical significance in the pattern of extracted metabolites i.e. 1D GC-TOFMS cell supernatant, *p* = 1.84*10^− 5^ (63 metabolites), 1D GC-TOFMS cell pellet, *p* = 0.03 (50 metabolites), 2D GC-TOFMS cell supernatant *p* = 0.0007 (180 metabolites), 2D GC-TOFMS cell pellet, *p* = 0.004 (122 metabolites). This analysis also revealed a large number of metabolite differences that were unrelated to the arginine metabolic pathway. This is shown in Tables 1 and 2 which summarizes all identified metabolites, their *p*-values and the metabolic pathway they belong to.

**Table 1:** Metabolites affected in the cell supernatant

Pathway	Metabolite	1D GC-MS	*p*-value	2D GC-MS	*p*-value	AA Analysis	*p*-value
Alanine And Aspartate Metabolism	Aspargine	↑	0.000			↑	0.002
	Alanine	↑	0.000			↑	0.007
	Aspartic Acid			↑	0.029	↓	0.135
Amino Sugar Metabolism	N-Acetyl Glucosamine	↑	0.000				
	N-Acetyl Mannosamine	↑	0.000				
Beta-Alanine Metabolism	Pantothenic Acid	↓	0.292	↑	0.009		
Citric Acid Cycle	Succinic Acid	↑	0.010				
Creatine Metabolism	Creatinine	↑	0.003				
	Cystein					↑	0.121
Cysteine And Methionine Metabolism	Cystein (2 Derivative)					↑	0.005
	Cystine	↑	0.005			↑	0.003
	Methionine	↑	0.003			↑	0.044
Fatty Acid Biosynthesis	3-Hydroxybutyric Acid			↑	0.004		
	Butanoic Acid			↑	0.000		
	Hexadecanoic Acid	↓	0.200				
	Fructose-1- Phosphate	↑	0.050				
Fructose And Mannose Degradation	Mannose	↓	0.020				
	Fructose	↑	0.011				
Galactose Metabolism	Galactose	↓	0.225				
Glutamate Metabolism	Glutamic Acid	↑	0.000			↑	0.247
	GABA					↑	0.001
	Glutamine	↑	0.000	↑	0.009		
Glutathione Metabolism	Pyroglutamic Acid	↑	0.000				
Glycerolipid Metabolism	Ethanolamine	↑	0.010				
Glycine, Serine And Threonine Metabolism	Glycine	↑	0.068	↑	0.012	↑	0.152
	Sarcosine	↑	0.000				
	Serine			↑	0.007	↑	0.020
	Threonine	↑	0.017			↑	0.155
Glycolysis. Gluconeogenesis.	Glucose	↓	0.143				
Pyruvate Metabolism	Glyceric Acid	↑	0.000				
	Lactic Acid	↑	0.002				
	Malic Acid	↑	0.039				
Histidine Metabolism	Histidine			↑	0.033	↑	0.027
Homocysteine Degradation	3-Methyl-2-Ketobutyric Acid			↑	0.000		
Inositol Metabolism	Inositol	↑	0.002				
Lysine Metabolism	Lysine	↑	0.044	↑	0.008	↑	0.191
	Lysine (2 Derivative)					↑	0.027
Nicotinate And Nicotinamide Metabolism	Nicotinamide	↑	0.021	↑	0.001		
	Arabinose	↑	0.003				
Nucleotide Sugar. Pentose Metabolism	Arabitol	↑	0.000				
Nucleotide Sugars Metabolism	Xylose	↑	0.004				
Pentose Phosphate Pathway	Gluconic Acid 1.4-Lactone	↑	0.034				
	Ribofuranose			↑	0.013		
	Ribose	↑	0.000	↑	0.028		
	Sedoheptulose-7-Phosphate	↑	0.276				
Phenylalanine Metabolism	Phenylalanine	↑	0.000	↑	0.022		
Purine Metabolism	Allantoin	↑	0.051				
Pyrimidine Metabolism	Uracil	↓	0.024				
Riboflavin Metabolism	Ribitol	↑	0.000				
Sugar. Sugar Substitute. Starch	Erythritol	↑	0.002				
Tryptophan Metabolism	Tryptophan					↑	0.035
Tyrosine Metabolism	Tyrosine	↑	0.034	↑	0.010	↑	0.052
Urea Cycle. Arginine and Proline Metabolism	Ammonia					↓	0.038
	Arginine	↑	0.015	↑	0.010	↑	0.070
(Arginine biosynthetic pathway)	Argininosuccinate	↑	0.021				
	Asymetrical-N.N- Dimethylarginine	↑	0.018				
	Citrulline					↑	0.098
	Citrulline (Arginine)	↑	0.016				
	Citrulline (Ornthine)	↑	0.019				
	Hydroxyproline			↑			
	Ornithine (2 Derivative)					↑	0.000
	Ornithine	↑	0.006			↑	0.156
	Proline	↑	0.000			↑	0.000
	Urea	↑	0.058	↑	0.001		
	2-Keto-3-Methylvaleric Acid			↑	0.002		
Valine. Leucine And Isoleucine Degradation	2-Oxoisocaproic Acid	↑	0.002	↑	0.000		
	Isoleucine	↑	0.008	↑	0.033	↓	0.373
	Leucine	↑	0.056			↓	0.253
	Valine	↑	0.010	↑	0.002	↓	0.132
Other	2-Aminobutyric Acid	↑	0.000				
	2-Deoxy-Galactose	↑	0.002				
	Allothreonine	↑	0.010	↑	0.000		
	Cellotriose	↓	0.224				
	Ellagic Acid	↓	0.293				
	Gluconic Acid	↓	0.095				
	Isoerythritol	↑	0.000				
	Mannitol	↓	0.031				
	Pentanoic Acid			↑	0.000		
	Propanoic Acid			↑	0.001		
	Silanamine			↑	0.021		
	Sorbose	↑	0.002				
	Xylitol	↑	0.000				

↑ Elevated in ASS+ve cell lines, ↓ lowered in ASS+ve cell lines. *p*-values was calculated using a Mann-Whitney U-test

**Table 2:** Metabolites affected in the cell pellet

Pathway	Metabolite	1D GC-MS	*p*-value	2D GC-MS	*p*-value	AA Analysis	*p*-value
Alanine And Aspartate Metabolism	Aspargine	↑	0.097			↑	0.077
	Alanine	↑	0.051	↑	0.274	↑	0.274
	Aspartic Acid			↑	0.068	↑	0.356
	Beta-Alanine	↑	0.511	↑	0.354		
Linoleic Acid Metabolism	Linoleic Acid			↑	0.324		
Amino Sugar Metabolism	N-Acetyl Glucosamine	↓	0.080				
Beta-Alanine Metabolism	Pantothenic Acid			↓	0.080		
Citric Acid Cycle	Alpha-Ketoglutaric Acid	↓	0.127	↓	0.047		
	Citric Acid			↓	0.263		
	Fumaric Acid			↑	0.329		
Creatine Metabolism	Creatinine	↓	0.165	↓	0.042		
	Cysteine	↓	0.113			↑	0.200
Cysteine And Methionine Metabolism	Methionine			↓	0.382	↓	0.619
Dipeptide	Glycylvaline	↑	0.168				
Fatty Acid Biosynthesis	Dodecanoic Acid	↓	0.649				
	Hexadecanoic Acid	↓	0.336				
	Mannose	↓	0.252				
Fructose And Mannose Degradation	Sorbitol-6-Phosphate (Fragment)	↑	0.600				
Galactose Metabolism	Galactose	↓	0,407				
Glutamate Metabolism	Glutamic Acid	↑	0.535			↑	0.195
	Glutamine	↑	0.651			↑	0.412
	GABA					↓	0.466
Glutathione Metabolism	Pyroglutamic Acid	↓	0.366	↓	0.125		
Glycerol Phosphate Shuttle	Glycerol-2-Phosphate	↑	0.184				
	Glycerol-3-Phosphate	↑	0.005	↑	0.003		
Glycerolipid Metabolism	Ethanolamine	↑	0.658				
	Serine			↓	0.081	↓	0.553
Glycine, Serine And Threonine Metabolism	Glycine	↓	0.487	↓	0.085	↓	0.157
	Threonine			↓	0.301	↑	0.033
	Sarcosine	↑	0.197				
Glycolysis, Gluconeogenesis,	Fructose-6-Phosphate	↑	0.103				
Pyruvate Metabolism	Glucose	↓	0.341				
Glycolysis, Gluconeogenesis,	Glucose-6-Phosphate	↑	0.174				
Pyruvate Metabolism	Glyceric Acid	↑	0.011				
	Glyceric Acid-2-Phosphate			↑	0.227		
	Glyceric Acid-3-Phosphate	↑	0.320	↑	0.228		
	Pyruvic Acid	↓	0.049	↓	0.000		
Homocysteine Degradation	3-Methyl-2-Ketobutyric Acid			↑	0.051		
Inositol Metabolism	Inositol	↓	0.066				
Lysine Metabolism	Lysine			↓	0.128	↑	0.404
	Lysine 2 Derivative					↑	0.128
	2-Amino-Adipic Acid			↑	0.043		
Nicotinate And Nicotinamide Metabolism	Nicotinamide			↓	0.371		
Nucleotide Sugar, Pentose Metabolism	Arabitol	↑	0.294				
Oxidative Phosphorylation	Pyrophosphate			↑	0.377		
Pentose Phosphate Pathway	Erythrose-4-Phosphate			↑	0.007		
	Glucaric Acid 1,4 Lactone	↓	0.622				
	Ribofuranose			↓	0.213		
	Ribose			↓	0.359		
	Ribose-5-Phosphate	↑	0.137				
Polyamine Metabolism	Putrescine	↓	0.653				
	Spermidine	↑	0.244				
Purine Metabolism	Adenine			↓	0.265		
	Hypoxanthine			↑	0.119		
Pyrimidine Metabolism	Uracil	↑	0.445				
	Uridine	↑	0.001				
Sugar, Sugar Substitute, Starch	Erythritol			↓	0.270		
Taurine And Hypotaurine Metabolism	Taurine					↑	0.282
Tryptophan Metabolism	Tryptophan					↑	0.104
Tyrosine Metabolism	Tyrosine			↓	0.221	↓	0.759
Urea Cycle, Arginine and	Ammonia					↑	0.258
Proline Metabolism	Arginine	↑	0.251			↑	0.502
(Arginine biosynthetic pathway)	Asymetrical-N,N-Dimethylarginine	↑	0.253				
	Citrulline (Arginine)	↑	0.247				
	Citrulline (Ornthine)	↑	0.193				
	Citrulline					↑	0.122
	N-Acetylornithine					↓	0.370
	Ornithine	↑	0.313	↑	0.219	↑	0.010
	2-Oxoisocaproic Acid	↑	0.600				
Valine, Leucine And Isoleucine Degradation	Isoleucine			↓	0.156	↓	0.550
	Leucine			↓	0.367		
	Valine					↓	0.085
Other	1,2-Ethandimine			↓	0.007		
	1,3,5-Trioxepane			↓	0.085		
	1-Mo nostearoylgly cer o l			↓	0.373		
	2-Pyrrolidone-5-Carboxylic Acid			↓	0.179		
	Aminomalonic Acid			↓	0.252		
	Cadaverine	↑	0.289				
	Cellotriose	↑	0.042				
	Dihydroxyacetonephosphate	↑	0.096	↑	0.195		
	Elaidic Acid			↑	0.165		
	Glucopyranose			↑	0.198		
	N-Acetyl Glutamyl Phosphate					↑	0.362
	Nonanoic Acid	↓	0.264	↓	0.144		
	Phosphoric Acid			↑	0.013		
	Pyrazine			↓	0.179		
	Stearic Acid			↑	0.357		
	Xylitol	↓	0.233				

↑ Elevated in ASS+ve cell lines, ↓ lowered in ASS+ve cell lines. *p*-values was calculated using a Mann-Whitney U-test

### Amino acid analysis

**Fig. 2:**
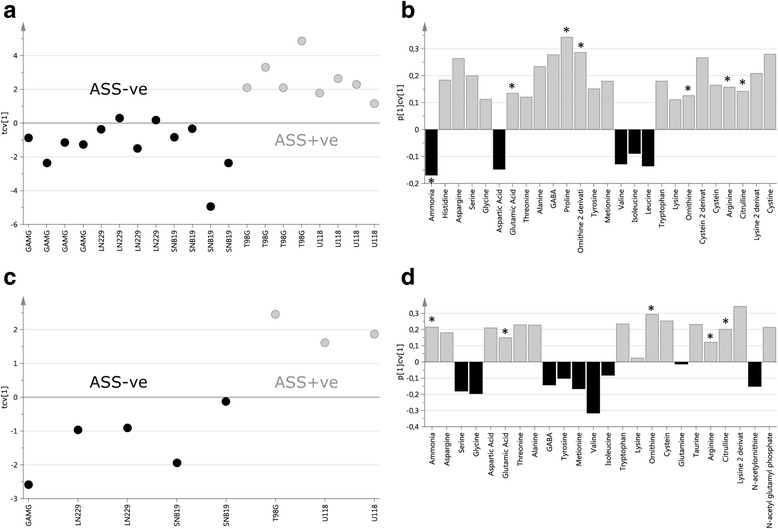
Cross-validated scores, first predictive score (tcv[1]) based on the final OPLS models for the amino acid analysis from (**a**) the cell supernatant and (**b**) corresponding loadings (p[1]cv[1]) from the cell supernatant and (**c**) cross-validated scores (tcv[1]) from the cell pellet and (**d**) corresponding loadings (p[1]cv[1]) from the cell pellet. The score plots show an almost complete separation between ASS+ve cell lines (grey) and ASS-ve cell lines (black). The loadings bar-plots show the metabolites responsible for the cell line separation. The metabolites marked in grey are elevated in the ASS+ve cell lines and metabolites marked in black are elevated in the ASS1-ve cell lines. The metabolites marked with * are involved in the arginine biosynthetic pathway, and elevated predominantly in ASS+ve cell lines

We next proceeded to validate some of the metabolites identified in our global screen using LC-MS by specifically targeting a selection of 29 amino acids. We included those involved in the arginine biosynthetic pathway primarily to determine if we could detect differences in this pathway between ASS positive and ASS negative cell lines, in line with our hypothesis. As well as successfully detecting all 29 amino acids by LC-MS, we were by means of OPLS-DA also able to detect metabolite differences that were related to the arginine biosynthetic pathway in these cell lines (Fig. 2). In the cell supernatant, glutamic acid, proline, ornithine, arginine and citrulline where elevated in ASS positive cell lines, all of which are included in the arginine biosynthetic pathway. Ammonia and leucine where elevated in the ASS negative cell lines. In the cell pellet, ammonia, glutamic acid, ornithine, arginine and citrulline were elevated in the ASS positive cell lines, all of which are included in the arginine biosynthetic pathway. Serine, glycine, GABA, tyrosine, methionine, valine, isoleucine and N-acetylornithine were elevated in the ASS negative cell lines.

The final cross-validated OPLS-DA score plot based on the pattern of these 29 amino acids showed the same class separation as detected using the global GC-TOFMS screening (supernatant *p* = 0.01, pellet *p* = 0.2).

### ASS positive and ASS negative GBM have different metabolic responses to arginine deprivation induced by ADI-PEG20 treatment

**Fig. 3:**
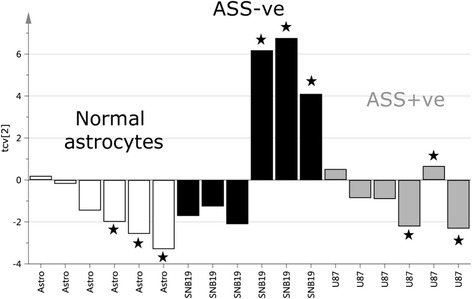
Cross-validated OPLS-DA scores, second predictive component (tcv[2]), for the GC-TOFMS data of cell supernatants of normal astrocytes (Astro; white bars), ASS-ve cells (SNB19; black bars) and ASS+ve cells (U87; grey bars). ADI-PEG20 treated samples are denoted by stars. The plot clearly shows that the ASS-ve samples are significantly affected by treatment (*p* < 0,001) while normal cells and ASS1 + ve cells remain largely unaffected

To investigate the metabolic response of these different GBM cell populations to arginine deprivation, a representative ASS negative (SNB19) and ASS positive (U87) cell line was treated with or without the arginine degrading enzyme, ADI-PEG20 (1 μg/ml) for 48 h and both supernatants and cell pellets were analysed by 1D GC-TOFMS. We included normal human astrocytes (non-tumour cells), and media alone in this analysis. The data generated from the 1D–GC-TOFMS analysis of the cell supernatants was subjected to OPLS-DA in order to obtain an overview of the metabolic variation between the samples. As can be seen in Fig. 3, the second predictive component, shows a unique effect in the ASS negative cells in response to ADI-PEG20 treatment (*p* = 0.001), while the ASS positive cells and normal astrocytes remain unchanged in that direction (metabolite signature). The first predictive component showed a difference associated with ADI-PEG20 treatment in astrocytes suggesting a general unspecific increase in metabolite concentration in those cells after treatment (not shown).

### Pathway and network analysis

**Fig. 4:**
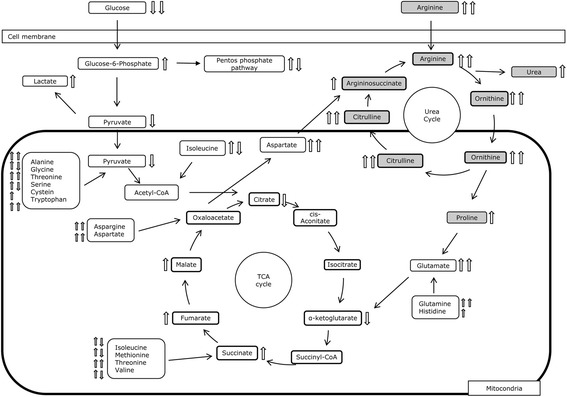
A summary of metabolite changes (1D GC/TOFMS data) between ASS+ve and ASS-ve cells in relation to biochemical mechanisms; metabolites in the Arginine biosynthesis pathway are coloured grey. The arrows describe the concentration change for the individual metabolites where ↑ indicate a metabolite higher in concentration in ASS+ve cells (lower in ASS-ve) and ↓ indicate a metabolite lower in concentration in ASS+ve cells (higher in ASS-ve). Open arrow represents cell supernatant and grey arrow cell pellet

To identify pathways that are most significantly altered between ASS negative and ASS positive cells, we performed pathway analysis using the IPA software of all the metabolite alterations detected using all three analytical methods. The analysis revealed the signalling and metabolic pathways and biological processes that are most significantly perturbed between these cells such as transfer of ribonucleic acid charging, super pathway of citrulline metabolism, arginine degradation VI, citrulline biosynthesis, arginine degradation I, proline biosynthesis II, urea cycle, glycine degradation, asparagine biosynthesis 1 and glutamate receptor signalling. These hits were consistent between all three datasets in the supernatant. In summary, these results indicate an alteration in citrulline and arginine metabolism between these two populations. Similar patterns were observed in the cell pellets with transfer ribonucleic acid charging being amongst the top 10 hits. Interestingly, large shifts in amino acid metabolism were observed in the extract, particularly with the amino acids alanine and glycine. The degradation of arginine in ASS negative cells to its respective products is decreased and α-ketoglutarate levels are concurrently increased, possibly due to lack of ornithine amino transferase activity. In the greater context of citrulline biosynthesis, the convergence of multiple pathways for citrulline production is observed. Decreases in both alanine and glutamate with corresponding increases in α-ketoglutarate and pyruvate suggests that ASS negative cells are converting less pyruvate to alanine which is one of the by-products of high glycolytic flux. There were clear changes in the metabolic intermediates generated by the breakdown of glutamine to fumarate in ASS negative versus ASS positive cells. Hence increased flux in these reactions may be due to increased levels of glutaminosis in ASS negative cells with glutamine being used to fuel the TCA cycle. A complete summary of the observed metabolite changes can be found in Fig. 4 and in Tables 1 and 2.

## Discussion

GBM is a fatal primary brain tumour for which there is no cure. Although numerous targeted therapeutic strategies have shown promise in pre-clinical models, none of these have successfully translated to the clinical setting.

We previously identified a new treatment option for a subset of primary GBM based on their inability to synthesize arginine, a semi-essential amino acid. We further showed that this inability was due to the transcriptional down-regulation of ASS1, the rate limiting step of arginine biosynthesis. This defect rendered GBM amenable to arginine deprivation therapy using the arginine degrading enzyme ADI-PEG20 [27]. Since ADI-PEG20 depletes blood arginine, this therapeutic strategy therefore potentially overcomes the limitations imposed by the blood brain barrier. An understanding of how ADI-PEG20 effects arginine levels in the CSF would help to advance our knowledge of how arginine deprivation affects the central nervous system and we plan to include such analysis in future clinical trials in GBM patients. Nevertheless, we have preliminary data showing regression of an intracranial GBM tumour in a xenograft mouse model after weekly intramuscular injections of ADI-PEG20 (manuscript in preparation).

Arginine is synthesized by the sequential action of two urea cycle enzymes, ASS1 and ASL. ASS1 converts citrulline and aspartate into argininosuccinate, with citrulline being derived from ornithine and recycled arginine and aspartate from the tricarboxylic acid (TCA) cycle. ASL then cleaves argininosuccinate into arginine and fumarate which can enter the TCA cycle. These enzymes thus participate in both the urea and TCA cycle. A deficiency in either gene will therefore lead to an accumulation of upstream metabolites i.e. citrulline and aspartate and a deficiency of downstream metabolites i.e. arginine and fumarate. Fumarate is an important intermediate in the TCA cycle for the generation of energy and arginine is a substrate for the generation of many metabolites that have key roles in numerous metabolic pathways which include cell signalling (NO, agamate), survival (NO) and proliferation (polyamines, proline). A deficiency in ASS1 would therefore have additional consequences on other pathways that rely on the substrates generated by ASS1 which may manifest an altered metabolic phenotype.

Since metabolomics allows for the global assessment of cellular states, this study was carried out to determine if ASS negative and ASS positive GBM have distinct basal metabolic phenotypes that could be exploited for their non-invasive detection in vivo and reveal new therapeutic strategies for the treatment of these GBM phenotypes. GBM is an extremely vascular and infiltrative tumour and complete surgical resection is often not possible. Since ADI-PEG20 has been shown to have anti-angiogenic effects, treating susceptible patients with ADI-PEG20 prior to surgery may help to reduce tumour growth and allow for more successful resection. It is important to note that the GBM cells used in this study were primary GBM cell lines (not having progressed from a lower grade counterpart) and not mutated for IDH1. IDH1 mutations are more common in low grade gliomas and in secondary GBM and predict longer survival [45]. However, 10% of primary GBM do present with this mutation, a feature that is known to influence the metabolome [46].

OPLS analysis of both the 1D and 2D GC-TOFMS data revealed clear systemic differences between these two populations of GBM cells. As hypothesized, an analysis of metabolites contributing to this difference identified an upregulation of those involved in the arginine biosynthetic pathway in ASS positive cells compared to their negative counterparts. Interestingly, these cells also exhibited an upregulation of numerous other metabolites which may be consistent with the fact that arginine is a substrate for many metabolites such as proline and creatine. This upregulation in metabolite levels was observed predominantly in the supernatant and not in the cell pellet where the distribution of elevated versus decreased metabolites was much more equivalent. In addition to mannose, galactose and glucose several other metabolites are decreased in the cell pellet in the ASS positive cell lines. Pyruvic acid, citrate and α -ketoglutaric acid, metabolites included in the initial steps of the citric acid cycle, are decreased in ASS positive cell lines.

Using pathway analysis to assess the biological significance of altered pathways between these two cell populations, it was clear that ASS negative cells have significantly altered arginine and citrulline metabolism in addition to large differences in amino acid metabolism.

This study provides methodological proof of concept for how metabolomics can be used in combination with other omics methodologies, in this case epigenetics, to generate a more detailed molecular characterization of GBM in terms of detecting and verifying specific molecular subgroups and suggest metabolic markers or rather marker patterns for the diagnosis and treatment of these GBM subgroups. It is a logical assumption that alterations in the genome or epigenome with effect on the phenotype would be reflected in the metabolome, which makes the characterization of the metabolome highly useful in order to filter out the relevant changes to the genome. In addition, altered metabolites or metabolic patterns are potentially markers for non-invasive diagnosis of new tumour subgroups, e.g. by magnetic resonance spectroscopy (MRS), and affected metabolic pathways can be used to reveal novel treatment targets for these. Our results show significant metabolite pattern changes between ASS positive and ASS negative cell lines and in relation to the specific treatment effect of arginine deprivation to ASS negative cells accentuate the strength of using metabolomics as a method to support and verify alterations in the epigenome. This highlights the possibility to develop and evaluate more efficient diagnostics and treatments within specific tumour types guided by more detailed molecular evidence as compared to the current standard, something that would not have been feasible without using the strategy of using metabolomics as a complement to one or more omics descriptions. An important part of this work is the bioinformatics approach based on chemometric or multivariate techniques which allowed us to extract metabolite patterns differentiating ASS positive and ASS negative cells as well as for the specific treatment effect in ASS negative cells. These metabolite patterns could potentially be refined into novel diagnostic markers, so called latent biomarkers, utilizing the strength in the correlation between co-varying metabolites as opposed to the traditional way of only considering single markers for diagnosis. From our results we could verify the differences between ASS positive and ASS negative cell lines related to the arginine biosynthesis pathway. However, our extracted metabolite pattern also included other significantly changing metabolites, something that could be of value both diagnostically as well as for pondering treatment options also for ASS positive tumours or other options for ASS negative tumours targeting other pathways. From a diagnosis point of view an interesting avenue to explore is the development of a non-invasive diagnosis of molecular tumour subgroups, in this case ASS positive and ASS negative GBM, by the use of MRS or similar techniques. If successful this would be a valuable contribution to the clinical practice. Interestingly, our differentiating metabolite pattern contained a number of metabolites detectable by MR spectroscopy in brain tumours (Tables 1 and 2), including glucose [47], glutamate [48], glutamine [49] and glycine [50]. Thus, it would be of high interest to investigate the diagnostic potential of such a metabolite pattern in vivo in an animal model, something that we aim to do in the near future. Another interesting prospect for the proposed methodology would be to do a more comprehensive screening of the GBM epigenome in combination with metabolomics analysis to construct a detailed map of the molecular subgroups of GBM that can be used to navigate towards improved diagnosis and development of tailored treatments based on molecular evidence.

## Conclusion

In conclusion we were able to verify our hypothesis that the metabolome contains systematic information discriminating between ASS1 positive and negative GBM cell lines and that there is a potential of identifying metabolite biomarkers for the non-invasive detection of these subtypes in addition to unveiling novel treatment targets. The study provides proof of concept for how metabolomics data combined with chemometric bioinformatics can be used to detect metabolite pattern changes, i.e. latent biomarkers, associated with alterations in the genome. Thus, providing a tool for detection of tumour subgroups based on specific molecular evidence.

## Abbreviations

ADI-PEG20: Pegylated arginine deiminase
ASL: Argininosuccinate lyase
ASS1: Argininosuccinate synthetase
DMEM: Dulbecco’s modified eagle medium
FCS: Fetal calf serum
GBM: Glioblastoma multiforme
GC-TOFMS: Gas chromatography-time-of-flight mass spectrometry
GCxGC: Two-dimensional gas chromatography
HCC: Hepatocellular carcinoma
IDH1: Isocitrate dehydrogenase 1
IPA: Ingenuity pathway analysis
LC-TOFMS: Liquid chromatography-time-of-flight mass spectrometry
MEM: Minimum essential media
MS: Mass spectrometry
OPLS-DA: Orthogonal partial least squares-discriminant analysis
PCA: Principal component analysis
PI3K: Phosphatidylinosintol 3 kinase
RB: Retinoblastoma
RTK: Receptor tyrosine kinase
