# Correction to: Metabolomic profiling identifies distinct phenotypes for ASS1 positive and negative GBM

**DOI:** 10.1186/s12885-018-4128-9

**Published:** 2018-03-08

**Authors:** Lina Mörén, Richard Perryman, Tim Crook, Julia K. Langer, Kevin Oneill, Nelofer Syed, Henrik Antti

**Affiliations:** 1 Department of Chemistry, Umeå University, SE 901 87 Umeå, Sweden; 2 John Fulcher Neuro-Oncology Laboratory, Imperial College London, London, UK; 3 St Luke’s Cancer Centre, Royal Surrey County Hospital, Guildford, Surrey UK

## Correction

In the original publication of this article [1], published on 8 February 2018, it was noticed that the acknowledgement of the source of the drug ADI-PEG20 was missing. In this Correction, the source of drug ADI-PEG20 is shown (and marked bold). This addition is made in the Methods section, under the heading ADI-PEG20 treatment.

ADI-PEG20 treatment.

SNB19 and U87 cells, cultured in DMEM + 10% FBS and normal human astrocytes, cultured in speciality media provided by lonza were seeded in replicates (*n* = 12) at 8 × 104 cells per well in 6-well dishes (Corning, NY, USA). 24 h post seeding, cells were washed with phosphate buffered saline (PBS) and cultured in the presence or absence of ADI-PEG20 **(kindly provided by Polaris Pharmaceuticals Inc, San Diego, CA)** (1 μg/ml) in media containing, 1 mM citrulline and 10% fetal FBS. ADI- PEG20 was added at the start of the experiment and no fresh media was added to any of the experimental plates before harvesting. ADI-PEG20 treated and untreated media (*n* = 3) was included for normalization purposes. 48 h after ADI-PEG20 treatment replicate samples for each condition (*n* = 3) were harvested, collecting both spent media and cells for GC-TOFMS metabolomic ana- lysis. Additional replicates (*n* = 3) of each condition were collected for total cell count determination.
